# High CD21 expression inhibits internalization of anti-CD19 antibodies and cytotoxicity of an anti-CD19-drug conjugate

**DOI:** 10.1111/j.1365-2141.2007.06883.x

**Published:** 2007-11-07

**Authors:** Gladys S Ingle, Pamela Chan, J Michael Elliott, Wesley S Chang, Hartmut Koeppen, Jean-Philippe Stephan, Suzie J Scales

**Affiliations:** 1 Departments of Molecular Biology South San Francisco, CA, USA; 2 Departments of Assay and Automation Technology South San Francisco, CA, USA; 3 Departments of Protein Chemistry South San Francisco, CA, USA; 4 Departments of Pathology, Genentech, Inc. South San Francisco, CA, USA

**Keywords:** CD19, CD21, CR2, immunoconjugates, non-Hodgkin lymphoma, antibody therapy

## Abstract

CD19 and CD21 (CR2) are co-receptors found on B-cells and various B-cell lymphomas, including non-Hodgkin lymphoma. To evaluate their suitability as targets for therapy of such lymphomas using internalization-dependent antibody-drug conjugates [such as antibody-4-(*N*-maleimidomethyl)cyclohexane-1-carboxylate, (*N*^2′^-deacetyl-*N*^2′^-(3-mercapto-1-oxopropyl)-maytansine) (MCC-DM1) conjugates, which require lysosomal degradation of the antibody moiety for efficacy], we examined uptake of antibodies to CD19 and CD21 in a panel of B-cell lines. Anti-CD21 antibodies were not sufficiently internalized even in the highest CD21-expressing Raji cells, resulting in lack of efficacy with anti-CD21-MCC-DM1 conjugates. Anti-CD19 antibody uptake was variable, and was unexpectedly negatively correlated with CD21 expression. Thus, high CD21-expressing Raji, ARH77 and primary B-cells only very slowly internalized anti-CD19 antibodies, while CD21-negative or low expressing cells, including Ramos and Daudi, rapidly internalized these antibodies in clathrin-coated vesicles followed by lysosomal delivery. Anti-CD19-MCC-DM1 caused greater cytotoxicity in the faster anti-CD19-internalizing cell lines, implying that the rate of lysosomal delivery and subsequent drug release is important. Furthermore, transfection of Ramos cells with CD21 impeded anti-CD19 uptake and decreased anti-CD19-MCC-DM1 efficacy, suggesting that CD21-negative tumours should respond better to such anti-CD19 conjugates. This may have possible clinical implications, as anti-CD21 immunohistochemistry revealed only approximately 30% of 54 diffuse large B-cell lymphoma patients lack CD21 expression.

Non-Hodgkin lymphoma (NHL) is one of the most rapidly increasing cancers in the United States, with approximately 63 000 new cases predicted for 2007 and a prevalence of approximately 360 000 (American Cancer Society, 2007). The most common subtype of NHL (representing approximately 30% of cases) is diffuse large B-cell lymphoma (DLBCL), while approximately 15% of cases involve the less aggressive follicular lymphoma (American Cancer Society, 2007). Most NHLs (85%) involve malignancies of the B-cells, many of which express the B-cell specific maturation antigen CD20 (Nadler *et al*, 1981). Rituximab (Rituxan®, Genentech, Inc., South San Francisco, CA, USA) is a chimeric anti-CD20 antibody that shows efficacy against B-cell lymphomas (James & Dubs, 1997) and has recently been approved as first line therapy in combination with chemotherapy for treatment of CD20^+^ NHLs (Cvetkovic & Perry, 2006). However, some lymphomas lack CD20 expression and a significant number of CD20^+^ patients do not respond to, or acquire resistance to, Rituximab therapy [reviewed by Smith (2003)], providing a rationale for investigating other targets and therapies for NHL.

One promising strategy for cancer therapy involves coupling cytotoxic drugs or radionucleotides to tumour-specific antibodies, thereby improving targeting to the tumour and decreasing non-specific toxicity compared with conventional chemo- or radiotherapy, as well as improving efficacy compared with naked antibody therapy (Vose, 1999; Polakis, 2005). Antibody-drug conjugates (ADCs) comprise tumour-specific antibodies chemically linked to cytotoxic drugs that are more potent than standard chemotherapeutics, resulting in excellent anti-tumour effects, but also systemic toxicity or ‘bystander’ effects (killing of nearby antigen-negative cells) if membrane-permeable drugs are released from the surface of cancer cells. Such release occurs when readily cleavable linkers are used, such as the reducible *N*-Succinimydyl 4-(2-Pyridyldithio) Pentanoate (SPP) linker (Xie *et al*, 2004; Austin *et al*, 2005; Kovtun *et al*, 2006) and acid-sensitive hydrazone linkers (Hamann *et al*, 2002; Doronina *et al*, 2003). To avoid non-specific toxicity, an ideal ADC would comprise a drug stably attached to the antibody such that the active drug were only released following internalization into the target cancer cell. One example is antibody-4-(*N*-maleimidomethyl)cyclohexane-1-carboxylate (MCC)- (*N*^2′^-deacetyl-*N*^2′^-(3-mercapto-1-oxopropyl)-maytansine) DM1 conjugates employing an uncleavable thioether succinimidyl-4-(*N*-maleimidomethyl)cyclohexane-1-carboxylate (SMCC) linker (which becomes MCC following conjugation) between the antibody and the maytansinoid microtubule polymerization inhibitor, DM1. However, these conjugates require efficient internalization and lysosomal degradation of the antibody to release the drug, which then diffuses within the cell and triggers cell death by preventing assembly of the mitotic spindle (Erickson *et al*, 2006). Antibodies to the B-cell receptor component CD79b internalize rapidly (within 20 min), being delivered to lysosomes within an hour and consequently anti-CD79b-MCC-DM1 conjugates show remarkable efficacy against CD79b-expressing xenografts (Polson *et al*, 2007). By contrast, CD20 antibodies are well-known not to internalize significantly even after prolonged incubation (Press *et al*, 1989, 1994; Sieber *et al*, 2003), so CD20 is not an ideal target for such ADCs with non-surface-cleavable linkers. The trafficking of anti-tumour antibodies following target binding clearly plays an important role in linker-drug selection.

CD19 (B4) has a wider expression profile than CD20 on both normal B-cells and NHL cells (Nadler *et al*, 1983; Uckun *et al*, 1988), and could be a more suitable ADC target as various anti-CD19 antibodies have been shown to internalize at different rates in several studies (Uckun *et al*, 1988; Press *et al*, 1989, 1994; Pulczynski *et al*, 1993; van Oosterhout *et al*, 1994; Sapra & Allen, 2002). However, other reports show no significant internalization (Ghetie *et al*, 1997; Cherukuri *et al*, 2001a; Sieber *et al*, 2003), and it is unclear whether this is due to use of different anti-CD19 antibodies, cell types or experimental conditions. CD19, in a complex with CD81 and CD21, acts as a co-receptor, enhancing signalling and antigen processing by the B-cell receptor in response to complement-tagged antigens (Fearon & Carroll, 2000). CD21 (also known as complement receptor 2 [CR2] or B2) is also associated with at least some B-cell lymphomas (Nadler *et al*, 1983; Scoazec *et al*, 1989; Gloghini & Carbone, 1993; Echeverri *et al*, 2002) but anti-CD21 antibody internalization has only been evaluated in a limited number of studies (Pulczynski *et al*, 1994; Tessier *et al*, 2006).

To evaluate the utility of both CD19 and CD21 as targets for antibody-MCC-DM1 conjugates, we examined the internalization of antibodies to these antigens by immunofluorescence in several malignant B-cell lines, as well as primary B-cells and correlated the uptake with sensitivity to the respective conjugates.

## Materials and methods

### Antibodies

Unless otherwise indicated, antibodies used were mouse anti-CD19 (clone B496, Biomeda CB-19; Biomeda, Foster City, CA, USA) and mouse anti-CD21 [HB135 (American Type Culture Collection, Manassas, VA, USA), also called THB-5 or HB5], anti-CD20 (2H7), anti-CD22 (RFB4) and anti-CD79b (SN8), all affinity purified at Genentech Inc. (South San Francisco, CA, USA) from hybridoma supernatants. Other anti-CD19 antibodies were BU12 (AnCell, Bayport, MN, USA), FMC63 (B19; Chemicon, Boronia, Vic., Australia) and HD37 (B4; Chemicon).

### Cell culture

Human B-cell lines were all cultured for a maximum of 2 months in RPMI medium, heat-inactivated 10% fetal bovine serum (FBS; Hyclone, Logan, UT, USA), 1%l-glutamine and were mycoplasma-free. Primary B-cells were isolated from normal human blood using the RosetteSep® non-B-cell depletion kit (StemCell Technologies, Vancouver, BC, Canada) according to the manufacturer’s instructions.

### CD21-Ramos generation

Two micrograms untagged CD21 full-length (isoform A, Swissprot 20023) subcloned into pCMV.PD5 using XbaI and BamHI (partial digest) was nucleoporated into 2 × 10^6^ Ramos cells in 100 μl Solution T and program O-06 according to the Amaxa Nucleofector II instructions. After 48 h recovery in a 12-well dish, cells were selected with 0·5 μg/ml puromycin (CellGro, Herndon, VA, USA) and 1% sodium pyruvate (Gibco, Carlsbad, CA, USA) for 18–24 d, after which high expressors were collected by flow cytometry with Alexa488-HB135 anti-CD21.

### Antibody uptake immunofluorescence

Cells were incubated for 5 min to 3 h in complete carbonate-independent medium (Gibco) in a 37°C waterbath (or in a 5% CO_2_ cell incubator in growth media for longer) with 1 μg/ml sterile test antibody, 1:100 human FcR block (Miltenyi Biotec, Auburn, CA, USA, dialyzed to remove azide), and 10 μg/ml Alexa488-transferrin or 25 μg/ml Alexa647-transferrin (Molecular Probes, Carlsbad, CA, USA) in the presence of 10 μg/ml leupeptin and 5 μmol/l pepstatin A (Roche Diagnostics, Indianapolis, IN, USA) to inhibit lysosomal degradation. Cells were then washed, fixed with 3% paraformaldehyde (Electron Microscopy Sciences, Hatfield, PA, USA), permeabilized with 0·4% saponin and the internalized antibody detected with 1 μg/ml Cy3 donkey anti-mouse Fc (Jackson Immunoresearch, West Grove, PA, USA), sometimes followed by anti-LAMP1 (Lysosomal Associated Membrane Protein-1) staining as previously described (Polson *et al*, 2007). Slides coverslipped with 4′,6-diamidino-2-phenylindole (DAPI)-containing Vectashield were viewed by epifluorescence microscopy with a DeltaVision® RT System (Applied Precision LLC, Issaquah, WA, USA), using a 100× Olympus UplanoApo objective. Images were captured with a Photometrics CH350 CCD camera powered by SoftWorx (version 3·4·4) software (Applied Precision LLC, Indianapolis, IN, USA) and assembled in Adobe Photoshop CS (Adobe Systems, Inc., San Jose, CA, USA).

### Surface flow cytometry

Cells were incubated with 2 μg/ml murine monoclonal antibodies to CD19 (B496, IgG1), CD21 (HB135, IgG2a), CD20 (2H7, IgG2a), CD22 (RFB4, IgG1), or CD79b (SN8, IgG1) with human FcR block in phosphate-buffered saline (PBS) containing 3% FBS on ice for 30 min, washed twice, then incubated with phycoerythrin (PE)-conjugated rat anti-mouse IgG1 or IgG2a + b (Becton Dickinson, Franklin Lakes, NJ, USA) for 30 min on ice. After two washes, cells were analyzed [with propidium iodide (PI) exclusion] on a FACSCalibur (BD Biosciences, San Jose, CA, USA).

### Quantitative uptake flow cytometry

Cells were incubated for 30 min at room temperature with 2 μg/ml Alexa488-anti-CD19, human FcR block and lysosomal protease inhibitors, shifted to 37°C for 20 min to 3 h, washed twice, and either fixed with 2% paraformaldehyde (PFA) or surface fluorescence quenched with 25 μg/ml rabbit anti-Alexa488 (Molecular Probes) for 1 h prior to fixation and analysis on a FACSCalibur (BD Biosciences). Uptake was calculated as previously described (Austin *et al*, 2004) and normalized to the amount of anti-CD19 initially bound. Where indicated, endocytic inhibitors were pre-incubated with cells for 30 min at 37°C at the following concentrations: 2 mmol/l methyl-β-cyclodextrin, 5 μg/ml filipin, 100 μmol/l chlorpromazine (all from Sigma Aldrich, St Louis, MO, USA), except for dynasore (TimTec, Newark, DE, USA), which was only pre-incubated with cells for 5 min at 80 μmol/l in serum-free media as reported (Macia *et al*, 2006). Background of inhibitor-treated cells without antibody was subtracted from the raw data prior to normalizing to the dimethyl sulphoxide (DMSO) control.

### Antibody-drug conjugates

Conjugates were synthesized as described (Polson *et al*, 2007), except that the antibodies used were the anti-CD21 clone HB135, anti-CD19 clone B496 and Trastuzumab anti-HER2 (Genentech Inc.). 7·5× molar excess SMCC was reacted with antibody for 4 h prior to DM1 addition, resulting in anti-CD21-MCC-DM1, anti-CD19-MCC-DM1 and Trastuzumab–MCC-DM1 conjugates with molar drug:antibody ratios of 4·05, 3·6 and 2·12 respectively.

### Cytotoxicity and apoptosis assay

Cells were seeded at 5000/well in 50 μl on clear round-bottomed 96-well plates and after 24 h were treated with serially diluted anti-CD19-MCC-DM1, anti-CD21-MCC-DM1, negative control Trastuzumab-MCC-DM1, naked antibody controls, or equivalent amounts of free L-DM1 dimer (serially diluted from 66·6 to 0·3 nmol/l), or normal growth medium at 37°C, 5% CO_2_. Cell viability and apoptosis were assessed after 3 and 2 d using the CellTiter Glo and Caspase Glo 3/7 kits, respectively, according to the manufacturer’s instructions (Promega, Madison, WI, USA).

### Immunohistochemistry

Sections (5 μm) of formalin-fixed, paraffin-embedded lymphoma tissue microarrays (Cybrdi, Frederick, MD, USA) on microscope slides were dewaxed and treated with Target Retrieval solution (Dako, Capinteria, CA, USA) at 99°C for 20 min in a boiling water bath. Endogenous peroxidase activity was quenched using 1× Blocking Solution (KPL, Gaithersburg, MD, USA) for 4 min. Sections were treated with Avidin/Biotin block (Vector) and Blocking Buffer containing 10% normal horse serum before sequentially incubating with 5 μg/ml anti-CD21 HB135, biotinylated horse anti-mouse (Vector), avidin-biotin peroxidase (Vector Labs, Burlingame, CA, USA), and biotinyl tyramide (Perkin-Elmer Inc., Waltham, MA, USA). Avidin-biotin peroxidase and diaminobenzidine (Pierce Biotechnology, Inc., Rockford, IL, USA) were used for detection.

## Results

### Anti-CD21 antibody HB135 is not significantly internalized

The expression of CD21 in human B-cell lymphomas is variable (Nadler *et al*, 1983; Echeverri *et al*, 2002), most likely in part because it is the receptor for Epstein–Barr virus (EBV), as infection with this virus induces high expression of CD21 (CR2) and its co-receptor CD35 (CR1; Freeman *et al*, 1982; Cohen *et al*, 1987). EBV-negative B-cell lymphoma lines, such as Ramos, SuDHL-4 and DoHH2, generally have undetectable levels of surface CD21, while EBV-positive cells like Daudi, ARH77 and Raji have higher levels (Table I).

**Table I:** Characteristics of cell lines used in this study (shown in order of increasing CD21 expression).

Cell line										
	Cell type	EBV	CD21*	CD19*	CD20†	CD22†	CD79†	CD77‡	CD19 uptake§	Naked effect¶
SuDHL-4	Diffuse large B-cell lymphoma	−	−	+++	+	+	+	ND	+++	++
Ramos	American Burkitt lymphoma	−	−	+++	+	+	+	++	+++	+
DoHH-2	Follicular lymphoma	−	−	+++	+	+	+	ND	+++	++
Namalwa	African Burkitt lymphoma	+	+	+++	+	+	+	−	++	ND
Daudi	African Burkitt lymphoma	+	+	+++	+	+	+	++	++	−
Ramos-CD21 clone 3	American Burkitt lymphoma +CD21^mod^	−	++	++	+	+	+	ND	++	+
ARH77	Plasma Cell leukemia	+	++	+++	+	+	−	−	−	++
Raji	African Burkitt lymphoma	+	+++	++++	+	+	−	+	−	−
Ramos-CD21 Clone 1	American Burkitt lymphoma +CD21^hi^	−	+++	++	+	+	+	ND	−	+

* Semi-quantitative FACS data from Fig 3. −, <5 mean fluorescent intensity units (MFI); +, 6–300 MFI; ++, 301–600 MFI; +++, 601–1200 MFI; ++++, >1201 MFI.

† FACS data not shown (+, antigen present at any expression level; −, antigen absent).

‡ Surface staining of globotriaosylceramide (also known as Gb3 or CD77) (+, present; −, absent; ND, not determined) according to Wiels *et al* (1981).

**Fig 1:**
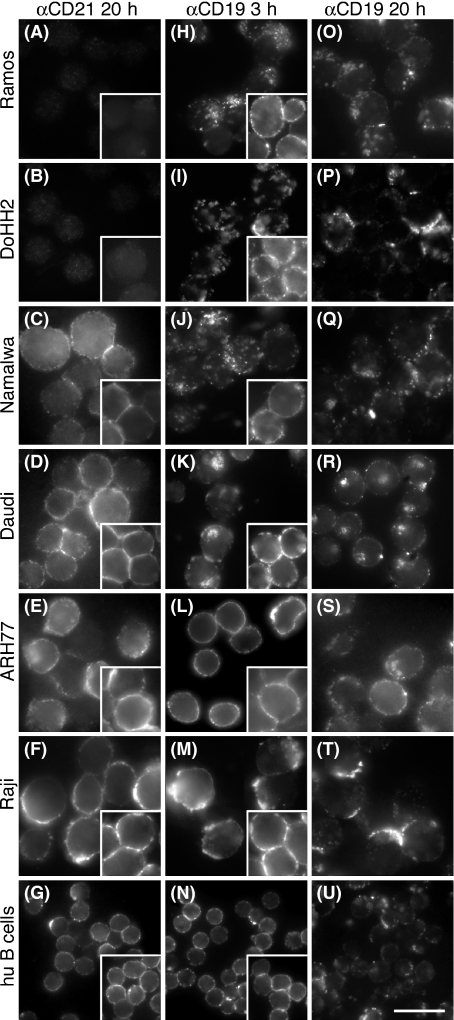
Anti-CD21 antibodies are not significantly internalized, while anti-CD19 antibodies only internalize readily in CD21^lo^ or CD21^−^ cells. Various B-cell lines were incubated with anti-CD21 (HB135) for 20 h at 37°C in the presence of lysosomal protease inhibitors, and the total antibody distribution detected post fixation and permeabilization with Cy3-conjugated anti-mouse (left panels). Insets show surface binding of anti-CD21 following 1 h incubation on ice. Ramos (A) and DoHH2 (B) cells lack surface expression of CD21 and consequently failed to internalize any antibody, as expected. Anti-CD21 is not significantly internalized in the low CD21-expressing Namalwa (C) or Daudi (D) cells, or even in the higher expressing ARH77 (E) or Raji (F) cells, or in freshly isolated primary human B-cells (G). The same cell lines were incubated with anti-CD19 (B496) antibodies on ice for 1 h (insets in middle panels), or at 37°C for 3 h (middle panels) or 20 h (right panels) with detection as above. The CD21-negative cell lines Ramos (H,O) and DoHH2 (I,P) readily internalized anti-CD19 within 3 h, while the low CD21-expressing Namalwa (J,Q) and Daudi (K,R) cells internalized it less extensively, as judged by the faint plasma membrane staining remaining even after 20 h uptake. The high CD21-expressors, ARH77 and Raji did not detectably internalize anti-CD19 after 3 h (L,M), and after 20 h still had not internalized nearly as much as the CD21-negative cells did in 3 h (S,T). Primary human B-cells did not internalize anti-CD19 within 3 h (N), but did by 20 h (U). Virtually all the cells in each field readily internalized Alexa488-transferrin (with the exception of transferrin-receptor negative primary B-cells), indicating that any lack of antibody uptake was not due to loss of viability (not shown). Gamma levels were adjusted where appropriate. Scale bar = 20 μm.

§ Anti-CD19 (B496) uptake from Fig 1 and data not shown. +++, extensive and rapid uptake detectable within 20 min; ++, extensive uptake within 3 h; − up to overnight incubation required to detect significant uptake.

¶ *In vitro* cytotoxic effect of naked B496 anti-CD19 data from Fig 5 and data not shown. −, no significant anti-proliferative effect *versus* control; +, ≤25% cell death *versus* control at optimal anti-CD19 concentration; ++, ≥26% cytotoxicity; ND, not determined.

The anti-CD21 antibody HB135 (abbreviated as ‘anti-CD21’ throughout) did not appreciably internalize in any B-cell line tested, irrespective of CD21 expression level (Fig 1A–F), even though the control co-incubated fluorescent transferrin was readily taken up (not shown). As expected, SuDHL-4 (not shown), Ramos and DoHH2 cells, which lack CD21 expression altogether, did not take up anti-CD21 (Fig 1A,B). However, even the moderately expressing Namalwa and Daudi cells (Fig 1C,D) and the higher expressing ARH77 and Raji cells (Fig 1E,F) failed to significantly internalize this antibody. Anti-CD21 similarly did not appreciably internalize in B-cells freshly isolated from normal human blood (Fig 1G), with antibodies mostly remaining at the cell surface similar to their distribution following incubation for 1 h on ice (insets), at which temperature all membrane traffic is inhibited. While removal of surface antibody signal by acid stripping did reveal a small amount of intracellular anti-CD21 antibody in Raji cells (data not shown), our experience with other B-cell specific antibodies showed that much more significant uptake (readily detectable without surface stripping) is required for effective drug delivery (Polson *et al*, 2007). The poor anti-CD21 uptake might explain the lack of efficacy of anti-CD21(HB135)-MCC-DM1 conjugates (which require good internalization and lysosomal delivery for drug release) even in the highest CD21-expressing Raji cells (see later) and ARH77 cells (data not shown). Taken together, these results suggest that CD21 is not a suitable target for ADCs requiring good cellular uptake for efficacy.

### Anti-CD19 antibodies only internalize significantly in CD21-negative or low cell lines

**Fig 2:**
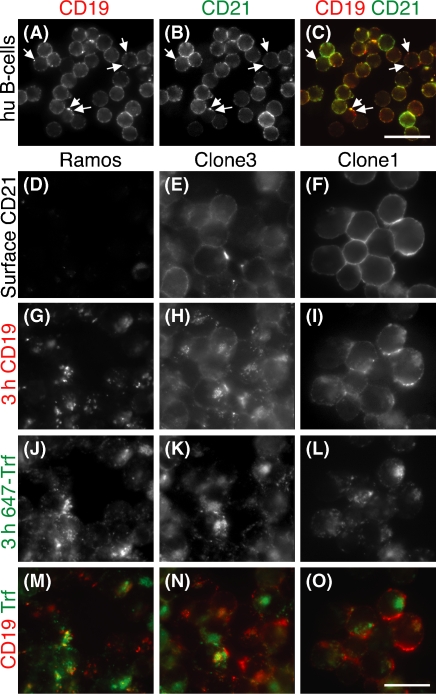
Transfection of CD21 into Ramos cells impedes anti-CD19 uptake. Upper panels: freshly isolated human B-cells do not internalize Alexa555-labeled anti-CD19 within 3 h (A, red channel in C), although it does redistribute into patches on the cell surface (arrows) that co-localize with Alexa488-labeled anti-CD21 added post-uptake on ice (B, green channel in C). Scale bar = 20 μm. Lower panels: Ramos cells (left panels) stably expressing a high (clone 1, right panels) or medium (clone 3, middle panels) level of CD21 were incubated with Alexa555-labeled anti-CD19 (G–I and red channel in M–O) and Alexa647-transferrin (J–L and M–O, shown in green channel for better contrast) for 3 h at 37°C, then chilled and incubated with Alexa488-conjugated anti-CD21 antibodies (D–F) on ice prior to fixation and imaging. Anti-CD19 uptake is impeded by increased CD21 expression, while transferrin uptake is unaffected. Internalized anti-CD19 antibodies do not significantly co-localize with the recycling transferrin at this time-point, as seen by lack of yellow colour in the respective overlaid images (M–O). Gamma levels were adjusted where required for clarity. Scale bar = 20 μm.

Internalization of various anti-CD19 antibodies has been reported in a number of B-cell lines and clinical samples with contradictory results (van Oosterhout *et al*, 1994; Press *et al*, 1994; Ghetie *et al*, 1997; Goulet *et al*, 1997; Sieber *et al*, 2003). Dimerization of anti-CD19 antibodies with a chemical linker (Ghetie *et al*, 1997), dimeric drug (Goulet *et al*, 1997) or cross-linking with secondary antibodies (Sieber *et al*, 2003) increases their uptake, but the latter is not relevant to ADC therapy, since secondary antibodies would not be present in the patient. We therefore focused on uptake in the absence of cross-linking of a novel anti-CD19 monoclonal B496 (henceforth abbreviated as ‘anti-CD19’), selected because of its stronger binding to Raji cells than other antibodies (data not shown, but see Fig S1). All the above B-cell lines bound significant amounts of this anti-CD19 antibody (insets in Fig 1H–N; see also FACS data below), but its rate of uptake varied widely and was not directly correlated to the CD19 expression level (Fig 1H–U) and Table I. Unexpectedly, it appeared instead to negatively correlate with CD21 expression level: the CD21-negative (CD21^−^) cell lines Ramos, DoHH2 and SuDHL-4 rapidly internalized anti-CD19 within 20 min (not shown), having internalized a significant amount by 3 h (Fig 1H,I and data not shown). By contrast, the high CD21 (CD21^hi^) expressors, ARH77 and Raji, showed no significant uptake within 3 h (Fig 1L,M) and only little uptake compared with the above cell lines after an overnight incubation (panels S,T). The low CD21-expressing (CD21^lo^) Namalwa and Daudi cells internalized anti-CD19 much better than the high CD21-expressors, but not as extensively as the CD21^−^ cells, as judged by the remaining plasma membrane staining after 3 h Fig 1J,K) and 20 h (Fig 1R). These results were not peculiar to the B496 antibody because Raji and ARH77 cells also only slowly internalized three other anti-CD19 antibodies, including the widely-used HD37 monoclonal (Fig S1A, B), which has been shown to internalize rapidly in Daudi cells (Press *et al*, 1989); while Ramos cells rapidly internalized HD37 and BU12 antibodies, and FMC63 to a lesser extent (Fig S1C). Primary human B-cells were also CD21^hi^ (Fig 1G), and did not appreciably internalize anti-CD19 within 3 h (Fig 1N), although the antibody did redistribute into patches on the cell surface along with CD21 (Fig 2A–C), similar to in Raji cells (Fig 1M), and did internalize to some extent within 20 h (Fig 1U).

### Expression of CD21 inhibits internalization of anti-CD19 antibodies

As CD21 is well-known to exist in a complex with CD19 (Fearon & Carroll, 2000) and CD21 is not significantly internalized (Fig 1), we hypothesized that CD21 bound to CD19 and prevented anti-CD19 antibodies from internalizing. To test this, we stably transfected CD21^−^ Ramos cells (Fig 2D) with CD21, obtaining a moderately expressing clone 3 (Fig 2E) and a highly expressing clone 1 (Fig 2F), as determined by surface labelling with anti-CD21 antibodies. We confirmed that the transfected CD21 was indeed complexed with endogenous CD19 by co-immunoprecipitation (Fig S2). In support of our hypothesis, anti-CD19 was well-internalized in Ramos cells in 3 h (Fig 2G), but less so in the moderately CD21-expressing clone 3 (Fig 2H) and even less so in the CD21^hi^ clone 1 (Fig 2I). This was not due to a general defect in endocytosis because transferrin appeared to internalize equally well in all three cell lines (Fig 2J–L) and uptake of antibody RFB4 against another B-cell antigen, CD22, was similarly unaffected (data not shown).

**Fig 3:**
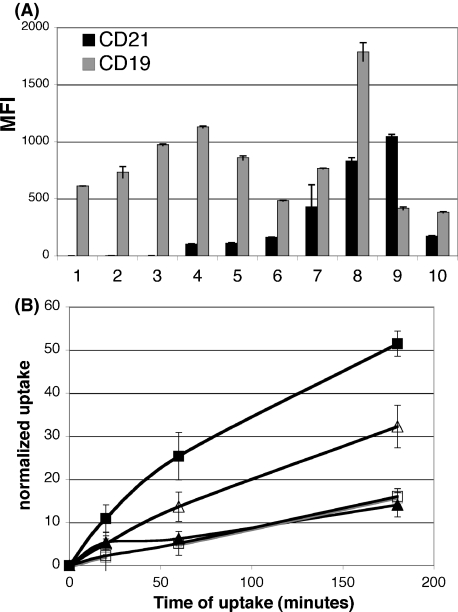
Quantitation of CD19 and CD21 surface levels and anti-CD19 uptake by flow cytometry confirms the immunofluorescence results. (A) B-cell lines were incubated on ice with 2 μg/ml mouse anti-CD21 (HB135) or mouse anti-CD19 (B496), followed by rat anti-mouse-phycoerythrin and analyzed by flow cytometry to determine surface expression. Results are the average mean fluorescence intensity (MFI) of triplicates ± standard deviation from a representative of three independent experiments (average of five independent experiments shown for the more variable ARH77 cells). Shown in increasing order of CD21 expression are: (1) SuDHL-4, (2) Ramos, (3) DoHH2, (4) Namalwa, (5) Daudi, (6) Ramos-CD21 clone 3, (7) ARH77, (8) Raji, (9) Ramos-CD21 clone 1. (10) Freshly isolated human B-cells have lower fluorescence values for both antigens than expected due to their small size, but their relative ratio of CD21 to CD19 is similar to that of ARH77 and Raji cells. Ramos-CD21 clone 1 expresses CD21 even more highly than Raji, while Ramos-CD21 clone 3 is intermediate between that of ARH77 and Daudi. (B) The rate of internalization of Alexa488-anti-CD19 in Ramos (▪), Ramos-CD21 clone 1 (□), Ramos-CD21 clone 3 (△) and CD21^hi^ ARH77 (▴) cells was determined by pre-binding to cells then incubating at 37°C (without washing) for the indicated times, washing and fixing either with or without surface fluorescence quenching with anti-Alexa488. Results are the average and standard deviation of two duplicate experiments each normalized to their respective initial surface binding levels after subtraction of background signals.

To confirm these results more quantitatively, we first compared the relative surface expression of both CD21 and CD19 on the panel of B-cell lines by flow cytometry (Fig 3A). Consistent with the immunofluorescence data, SuDHL-4, Ramos and DoHH2 cells completely lacked CD21 surface expression; Namalwa and Daudi had low expression of CD21; and ARH77 and Raji had higher expression. CD21 levels decreased with time since passaging and culture age, most likely due to shedding of the HB135 epitope, as previously documented in Raji cells (Fremeaux-Bacchi *et al*, 1998), but the overall trend of expression across the cell lines remained the same. ARH77 cells were particularly variable over time, sometimes having CD21 levels as high as 88% of the levels in Raji cells, hence their designation as high expressors. Ramos-CD21 clone 3 expressed CD21 at a level intermediate between those of ARH77 and Daudi, while clone 1 expressed CD21 even more highly than Raji. Furthermore, while absolute molecule numbers were not determined, Ramos-CD21 clone 1 was the only cell line with relatively higher (approximately 2·5-fold) apparent binding of anti-CD21 than anti-CD19. As expected, all the B-cell lines highly expressed CD19, especially Raji, although both Ramos-CD21 clones had slightly lower CD19 expression than the parental Ramos cells. By comparison primary human B-cells exhibited lower CD19 and CD21 FACS shifts than expected from their immunofluorescence intensity (Fig 2A,B), most likely due to their significantly (approximately 3×) smaller surface area for antibody binding than cultured B-cell lines (compare cell sizes in Fig 1G,F). However, the relative apparent ratio of CD21 to CD19 in primary B-cells was similar to those of ARH77 and Raji cells (>0·4), in agreement with the slow uptake of anti-CD19 antibodies in these cells (Fig 1N,U).

We next compared the rates of uptake of Alexa488-labeled anti-CD19 in the two Ramos-CD21 clones, quenching any remaining surface signal with anti-Alexa488 antibodies after different times to measure internalized anti-CD19 by flow cytometry and normalizing the data to their relative expression levels (Fig 3B). CD21^−^ Ramos cells internalized over half the initially bound anti-CD19 antibody within 3 h, about four times faster than the CD21^hi^ ARH77 cells. Ramos-CD21^hi^ clone 1 internalized anti-CD19 at a similar low rate to ARH77 cells, while the more moderately expressing clone 3 internalized at an intermediate rate, in agreement with the immunofluorescence data.

### Anti-CD19 is internalized via clathrin-dependent endocytosis and is delivered to lysosomes

**Fig 4:**
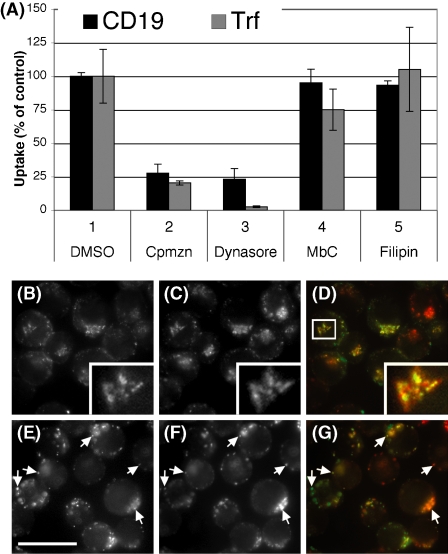
Anti-CD19 is internalized by dynamin-dependent, clathrin-mediated endocytosis and is delivered to lysosomes. (A) Ramos cells were pre-incubated for 30 min at 37°C with the following reagents: dimethyl sulphoxide (DMSO) (1); 1 μmol/l chlorpromazine (Cpmzn) (2), a clathrin-mediated endocytosis inhibitor; 80 μmol/l dynamin inhibitor dynasore, preincubated for 5 min only (3); 2 mmol/l methyl-β-cyclodextrin (MbC) (4) or 5 μg/ml filipin (5), both inhibitors of caveolar and lipid raft endocytosis. Alexa488-anti-CD19 (black bars) or Alexa488-transferrin (grey bars) were then added in the continuous presence of inhibitors for 30 min and surface quenched as in Fig 3B. Results were plotted as a percentage of uptake compared with the DMSO control and represent the average and standard deviation of three independent triplicate experiments. (B–D) Alexa488-anti-CD19 (green channel in B and D) was co-internalized with Alexa647-transferrin (shown in the red channel in C and D) in Ramos cells for 5 min, surface quenched with anti-Alexa488, fixed and imaged. (E–G) Alexa488-anti-CD19 (green channel in E and G) was chased for 3 h in Ramos cells in the presence of lysosomal protease inhibitors prior to fixation and staining with Alexa555-anti-LAMP1 (red channel in F and G). Yellow colour in the merged images in panels D and G indicates colocalization. Gamma levels were adjusted where necessary to better illustrate marker overlap. Arrows indicate examples of co-localized staining. Scale bar is 20 μm in the main panels and 6·7 μm in the 3×-magnified insets of the boxed region indicated in D.

The efficacy of antibody-MCC-DM1 conjugates depends not only on internalization, but also on effective delivery to lysosomes, permitting antibody degradation and DM1 metabolite release (Erickson *et al*, 2006). We therefore investigated the endocytic pathway taken by anti-CD19 antibodies in CD21^−^ Ramos cells. Alexa488-labeled anti-CD19 uptake after 30 min of continuous incubation was quantified in cells pre-treated with various endocytic inhibitors and compared with Alexa488-transferrin, which is well established to internalize via clathrin-mediated endocytosis prior to recycling (Watts & Marsh, 1992). Dynamin is a GTPase involved in the fission of both clathrin-coated and caveolar vesicles (Schmid *et al*, 1998), whose activity can be curbed by the novel small molecule inhibitor, dynasore (Macia *et al*, 2006). Internalization of both anti-CD19 and transferrin was dramatically inhibited by both dynasore and the clathrin inhibitor chlorpromazine, implicating clathrin-mediated endocytosis in anti-CD19 uptake (Fig 4A).

By contrast, the lipid raft and caveolar uptake inhibitors methyl-β-cyclodextrin and filipin (Fig 4A) had little effect on uptake of either anti-CD19 or transferrin, in line with the lack of caveolin in B-cells (including Ramos, data not shown) and lymphomas (Fra *et al*, 1994) and the reported lack of redistribution of CD19 into lipid rafts upon antibody cross-linking (Petrie *et al*, 2000). While the poor transfection efficiency of Ramos cells precluded confirmation of these results using biological inhibitors, such as dominant negative dynamin or Rab GTPase constructs, we were able to demonstrate colocalization of Alexa488-anti-CD19 with transferrin during the first 5 min of uptake (rendered visible by quenching the surface signal with anti-Alexa488 antibodies; Fig 4B–D), consistent with co-internalization in clathrin-coated vesicles. By 20 min of chase, anti-CD19 had started to diverge from the transferrin recycling pathway and by 60 min, partially overlapped with LAMP1^+^ late endosomes and lysosomes (data not shown), more extensively so after 3 h (Fig 4E–G), consistent with the poor co-localization with transferrin seen by this time-point (Fig 2M–O). Anti-CD19 antibodies were also delivered to lysosomes by 3 h in all the other CD21^lo/−^ internalizing B-cell lines examined (Fig S3 and data not shown).

### CD21 expression decreases the *in vitro* efficacy of anti-CD19-MCC-DM1 conjugates

**Fig 5:**
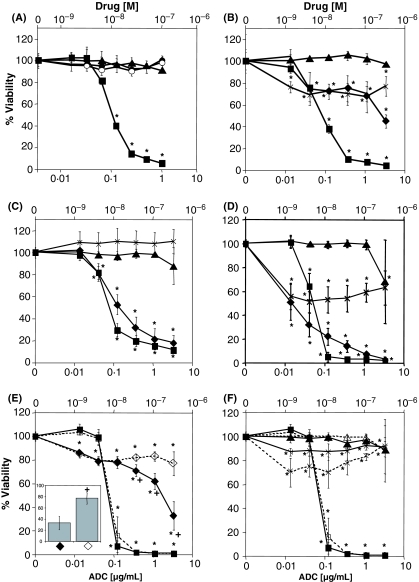
Anti-CD19-MCC-DM1 is less efficacious in high CD21 expressing cells. (A) CD21^hi^ Raji were incubated for 3 d with anti-CD21-MCC-DM1 (○), negative control Trastuzumab-MCC-DM1 (▴), free L-DM1 dimer (▪) or naked anti-CD21 antibodies (•) and assessed for viability by measuring ATP levels. CD21^hi^ ARH77 (B), CD21^lo^ Daudi (C), and CD21^−^ DoHH2 cells (D) were incubated for 3 d with anti-CD19-MCC-DM1 (♦), negative control Trastuzumab-MCC-DM1 (▴), free L-DM1 dimer (▪) or naked anti-CD19 antibodies (**×**) and assessed for viability by measuring ATP levels. (E) Ramos (solid symbols and lines) and Ramos-CD21 clone 1 (open symbols and dashed lines) were treated with anti-CD19-MCC-DM1 (♦,⋄) or free DM1 (▪,□) as in B-D. Inset bar graph shows percentage killing of Ramos (♦) and Ramos-CD21 clone 1 (⋄) at the highest anti-CD19-MCC-DM1 concentration used (3·33 μg/ml). (F) Ramos (solid symbols and lines) and Ramos-CD21 clone 1 (open symbols and dashed lines) were treated with control Trastuzumab-MCC-DM1 (▴,△), free DM1 (▪,□) or unconjugated anti-CD19 (**×**) as in B. Data are shown in all panels as a percentage viability of untreated control cells (mean and standard deviation of three independent duplicate experiments) *versus* ADC concentration in μg/ml on the lower *x*-axes or free DM1 concentration in M on the upper *x*-axes. * denotes data points statistically different (*P* < 0·01) from the control untreated cells using the analysis of variance (anova) test. +, data points significantly different between Ramos and Ramos-CD21 clone 1 cells by anova analysis (*P* < 0·01). The CD21^hi^ Raji and Ramos-clone 1 cells showed greater resistance (compared with their respective free L-DM1 sensitivities) than the CD21^−^ Ramos, DoHH2 and CD21^lo^ Daudi cells.

Having established that internalized anti-CD19 antibodies end up in lysosomes, we examined whether the inhibition of uptake of anti-CD19 antibodies by CD21 affected the efficacy of the anti-CD19-MCC-DM1 ADC. Since the cytotoxic effect of DM1 is mediated by preventing the assembly of the mitotic spindle, cells were incubated with different concentrations of the ADC for at least two to three cell divisions (3 d) to assess its anti-proliferative effect compared with the free drug. CD21^hi^ ARH77 cells showed almost no greater response to anti-CD19-MCC-DM1 than to an equivalent concentration of naked anti-CD19 antibody alone (Fig 5B), suggesting that the ADC *per se* had little effect in this slowly internalizing cell line.

The anti-proliferative effect of naked anti-CD19 was small but statistically significant, albeit somewhat unexpected, as most other naked anti-CD19 antibodies (BU12, HD37 and FMC63) are reportedly without effect in several B-cell lines (Chaouchi *et al*, 1995; Sapra & Allen, 2002; Flavell *et al*, 2006) unless cross-linked or dimerized (Ghetie *et al*, 1997). Light scatter analysis confirmed that our anti-CD19 antibody was devoid of detectable aggregates or dimers (data not shown). Possible reasons for this discrepancy are that we used lower concentrations of anti-CD19, or that our ATP detection assay is more sensitive than the above studies, since BU12 and HD37 (but not the weaker binding FMC63 antibody) caused similar growth inhibition to our B496 antibody (Fig S4A). In agreement with previous studies using HD37 at higher concentrations (Ghetie *et al*, 1994), this was not due to apoptosis, since none of the naked antibodies significantly stimulated caspase 3/7 activity within 48 h, although free DM1, and to a lesser extent anti-CD19-MCC-DM1, did (Fig S4B).

In contrast to the lack of effect of anti-CD19-MCC-DM1 in ARH77 cells, this ADC was much more effective in CD21^lo^ Daudi cells (Fig 5C), even though these cells were no more sensitive to free DM1. Cytotoxicity was specific because anti-CD19-MCC-DM1 did not affect proliferation of DM1-sensitive, CD19-negative Jurkat cells (Fig S4C) and Trastuzumab-MCC-DM1 (whose target, HER2, is not expressed on any B-cell lines) had no significant effect any of the cell lines. The faster internalizing CD21^−^ DoHH2 cells were even more sensitive to anti-CD19-MCC-DM1 than free DM1, although this could be partly due to the pronounced anti-proliferative effect of the naked antibody in this cell line (Fig 5D). More importantly, Ramos-CD21 clone 1 cells were significantly less responsive to CD19-MCC-DM1 than Ramos cells (Fig 5E), despite having similar growth rates and similar sensitivities to both the free drug and the naked anti-CD19 antibody (Fig 5F). Specifically at 3·3 μg/ml anti-CD19-MCC-DM1 conjugate, 67% of Ramos cells were dead compared with only 23% of Ramos-CD21 clone 1 (inset in Fig 5E). In fact, most, if not all of the cytotoxicity in Ramos-CD21 cells may be attributable to the effect of naked anti-CD19 (compare Fig 5E with F), similar to the situation in ARH77 cells (Fig 5B). As expected, anti-CD19-SPP-DM1, which employs a surface cleavable (reducible) SPP linker (Austin *et al*, 2005) and so does not depend on lysosomal delivery for efficacy, did not show any significant difference in killing between the Ramos and Ramos-CD21 cells (data not shown). The rate of anti-CD19 antibody uptake therefore does correlate with the efficacy of the anti-CD19-MCC-DM1 conjugate *in vitro*, and implies that such conjugates may be more effective at treating CD21^−^ or CD21^lo^ CD19^+^ tumours *in vivo.*

### CD21 expression in CD19^+^ lymphomas is variable

While it remains to be determined if the enhanced cytotoxicity of anti-CD19-MCC-DM1 in the CD21^lo/−^ lines translates to greater efficacy in preclinical models, we sought to estimate the level of CD21 expression in patient samples. Previous reports suggest up to two-third of B-cell lymphomas express CD21, but little distinction was made between low and high expressors (Nadler *et al*, 1983; Scoazec *et al*, 1989; Gloghini & Carbone, 1993; Echeverri *et al*, 2002; Otsuka *et al*, 2004). Using anti-CD20 to confirm the identity of not otherwise specified B-cell lymphomas (BCL), we scored the expression of CD21 as negative (−), low (1+), or high (2+ or higher). Twenty-nine per cent of 24 CD20^+^ (and therefore presumably CD19^+^; none of the anti-CD19 antibodies recognized fixed specimens to confirm this directly) BCL patients lacked detectable CD21 staining in neoplastic B-lymphocytes; another 29% had only 1+ expression; and 42% had high expression (Table IIA), which is reasonably consistent with published results using the anti-B2 antibody to CD21 (Nadler *et al*, 1983). Similarly, in a set of 54 diffuse large B-cell lymphoma (DLBCL) cases, 28% of CD20^+^ DLBCL samples lacked detectable CD21, a further 33% had only low expression, and 39% had high expression (Table IIA), confirming that CD21 expression in clinical specimens is indeed variable. Furthermore, in a limited number of frozen DLBCL, low-grade NHL and follicular lymphoma specimens (in which the anti-CD19 epitope is preserved), we were able to confirm by dual label immunofluorescence (Fig S5) that CD21 was co-expressed in the same cells as CD19 in approximately two-thirds of cases, with lower intensity in approximately half of those, and no expression in the remaining one-third of cases (Table IIB).

**Table IIB:** Dual immunofluorescence (IF) analysis of CD19 and CD21 expression in frozen lymphoma specimens.

Lymphoma type	CD21^−^*	CD21 1+†	CD21 ≥ 2+‡	CD21 < CD19§
DLBCL^a^	3/7 (43)	1/7 (14)	3/7 (43)	3/7 (43)
Low grade NHL	3/7 (43)	2/7 (29)	2/7 (29)	4/7 (57)
Follicular lymphoma	1/3 (33)	2/3 (67)	0/3 (0)	2/3 (67)

Values are expressed as *n* (%).

DLBCL, diffuse large B-cell lymphoma; NHL, Non-Hodgkin lymphoma.

* CD21−, CD21 staining is absent from neoplastic CD19^+^ B-lymphocytes (an example of such a specimen is shown in Fig S5B).

† 1+, weak CD21 expression in CD19^+^ B-lymphocytes (an example being shown in Fig S5C).

‡ At least 2+, 2+ or 3+ expression of CD21 in CD19^+^ B-lymphocytes (see example in Fig S5D).

§ CD21 < CD19, CD21 IF score is lower than CD19 IF score (e.g. Fig S5C). Note that the different antibody affinities were not taken into account for this analysis; CD21 and CD19 levels were independently scored as negative to 3+ across all the tumour samples, then the final IF scores for the two antibodies were reviewed.

**Table IIA:** Immunohistochemical analysis of CD21 expression in CD20^+^ B-cells of formalin-fixed, paraffin-embedded lymphoma samples.

Lymphoma type	CD21^−^*	CD21 1+†	CD21 ≥ 2+‡
BCL	7/24 (29)	7/24 (29)	10/24 (42)
DLBCL	15/54 (28)	18/54 (33)	21/54 (39)

Values are expressed as *n* (%).

BCL, not otherwise specified B-Cell lymphoma; DLBCL, diffuse large B-cell lymphoma.

* CD21^−^, CD21 staining is absent from neoplastic CD20^+^ B-lymphocytes.

† 1+, weak CD21 expression in CD20^+^ B-lymphocytes.

‡ ≥2+, high 2+ or 3+ expression of CD21 in CD20^+^ B-lymphocytes.

## Discussion

We have shown that B-cell lines do not appreciably internalize the HB135 anti-CD21 antibody, even when CD21 is highly expressed, as in ARH77, Raji and primary human B-cells. This most likely explains the lack of effect of anti-CD21-MCC-DM1 (Fig 5A), since internalization is required for drug release from SMCC-DM1 conjugates (Erickson *et al*, 2006; Polson *et al*, 2007). Our data are consistent with the lack of anti-CD21 internalization in B-cells observed in an earlier study (Barrault & Knight, 2004), although internalization of CD21 could be induced by binding of its ligands, C3d or EBV (Tedder *et al*, 1986; Perrin-Cocon *et al*, 2004) or when it is expressed in the absence of CD19 and the B-cell receptor in 293 cells (Tessier *et al*, 2006).

We also found that the internalization of four different anti-CD19 antibodies was interestingly negatively correlated with CD21 expression such that CD21^hi^ Raji, ARH77 and normal B-cells failed to significantly internalize, while cells with low CD21, i.e. Daudi, Namalwa (also Granta, data not shown), or no CD21, i.e. Ramos, DoHH2 (and SuDHL-4, data not shown), internalized anti-CD19 rapidly (within 5–20 min). Conversely, siRNA-mediated knockdown of CD21 in Raji cells permitted anti-CD19 uptake in this cell line (data not shown). There was no correlation of anti-CD19 uptake with the other major B-cell markers, CD20, CD22 or CD79b (Table I); nor did CD21 expression correlate with uptake of antibodies to any of those proteins (data not shown). Since at steady-state CD19 was only detectable at the cell surface by immunofluorescence (data not shown), it is likely that the anti-CD19 antibodies trigger internalization of CD19 in CD21^lo/−^ cells by cross-linking of CD19 to itself, rather than ‘catching a ride’ on constitutively internalizing CD19. Significantly, Ramos cells transfected with CD21 no longer displayed such rapid uptake of anti-CD19 antibodies, presumably because CD21 forms a non-covalent complex with CD19 (Tedder *et al*, 1994; Fearon & Carroll, 2000), as shown by immunoprecipitation. One explanation could be that complex formation prevents self cross-linking of CD19 by anti-CD19 antibodies, retarding their internalization. Note that anti-CD19 and anti-CD21 antibodies (including B496 and HB135, data not shown) can be made to internalize in CD21^hi^ Raji and ARH77 cells by cross-linking with anti-mouse secondaries (Pulczynski *et al*, 1993; Sieber *et al*, 2003), but this is probably irrelevant to antibody-MCC-DM1 therapy, as the antibodies would be humanized prior to clinical trials and anti-human antibodies would not be present in the patients. Even so, consistent with our hypothesis, Pulczynski *et al* (1993) demonstrated faster internalization of cross-linked anti-CD19 (B4) in the CD21^−^ pre-B blast cell line NALM6 than in CD21^hi^ Raji cells, despite these cell lines having similar CD19 levels.

Our data are in agreement with the observed uptake of various other anti-CD19 antibodies in CD21^−^ NALM-6 and CD21^lo^ Daudi and Namalwa cells (Nadler *et al*, 1983; Uckun *et al*, 1988; Press *et al*, 1989; Goulet *et al*, 1997; Sapra & Allen, 2002). Our results may also explain the lack of anti-CD19 uptake in B-cells from patients with B-cell chronic lymphocytic leukemia (B-CLL) (Sieber *et al*, 2003), since these are mostly CD21^+^ (Nadler *et al*, 1983; Lopez-Matas *et al*, 2000; Cherukuri *et al*, 2001a). Furthermore, antibodies to both components of the CD19/CD21 complex were found not to internalize in murine B-cell lymphoma CH27 or splenic B-cells even after cross-linking to antigen or complement (Cherukuri *et al*, 2001a, b). We took precautions to avoid any CD19-independent uptake of anti-CD19 by Fc-γ receptors (as measured by Vervoordeldonk *et al* (1996)) by including human Fc-receptor block in all our experiments.

The actual endocytic pathway responsible for anti-CD19 uptake in all these studies has not been characterized, with the exception of its visualization by electron microscopy in unidentified plasmalemmal pits and eventually in lysosomes following secondary antibody-mediated cross-linking in Raji and NALM-6 cells (Pulczynski *et al*, 1993). We found that, in the absence of CD21, non-cross-linked anti-CD19 most probably internalizes via clathrin-mediated endocytosis, based on its colocalization at early time-points (5 min) with the clathrin cargo transferrin and inhibition of uptake of both proteins with the clathrin inhibitor chlorpromazine. Consistent with this, CD19 has two potential tyrosine-based internalization motifs (YEDM and YENM) in its cytoplasmic tail that could be predicted to bind the clathrin adaptor AP-2 (Bonifacino & Traub, 2003). In CD77 (globotriaosylceramide)-positive Daudi cells, cross-linked anti-CD19 was found mainly in the endoplasmic reticulum and nuclear envelope (Khine *et al*, 1998), but our non-cross-linked anti-CD19 showed no such localization, being transported instead to late endosomes and lysosomes within 1–3 h. Furthermore, antibody uptake showed no correlation with CD77 status (Table I), with CD77^−^ Namalwa cells showing very similar uptake kinetics and antibody distribution in lysosomes to CD77^+^ Daudi cells (which express similar CD21 levels).

The observed decrease in anti-CD19 antibody internalization by expression of CD21 also resulted in decreased sensitivity to the anti-CD19-MCC-DM1 ADC, confirming earlier results that efficient lysosomal delivery is required for activity of antibody-MCC-DM1 conjugates (Erickson *et al*, 2006; Polson *et al*, 2007). As the antibody must be degraded in lysosomes to release the free drug (or MCC-DM1 linked to the conjugating lysine in this case; Erickson *et al*, 2006), we propose that a rapid rate of lysosomal delivery is probably required to ensure a sufficient concentration gradient of released drug to diffuse across the lysosomal membrane into the cytoplasm and reach the target microtubules. In the case of the slowly internalizing CD21^hi^ cells, lysine-MCC-DM1 is probably not released quickly enough from anti-CD19 to generate a sufficient diffusion gradient for effective microtubule inhibition and cytotoxicity, most likely being inactivated in the lysosomes instead.

The low level of anti-proliferative activity of anti-CD19-MCC-DM1 in Ramos-CD21 clone 1 cells was partially, if not mostly, due to a naked antibody effect, which, unlike the MCC-DM1 conjugate, was less effective at high concentrations for reasons that are unclear. This naked anti-CD19 effect was unrelated to both CD21 and CD19 expression levels, occurring most markedly in DoHH2, SuDHL-4 and ARH77 cells, less so Ramos (and Ramos-CD21) and not at all in Raji or Daudi cells (summarized in Table I). At least one other naked anti-CD19 antibody (HB-12b) has been previously shown to inhibit proliferation of other B-cell lines Arent and OCI-LY8, expressing different levels of CD19 (Bradbury *et al*, 1992).

Our immunohistochemical results show that approximately one-third of DLBCL (and also unclassified BCL) patients lack CD21 expression, with another approximately one-third having low CD21 expression, in agreement with earlier analyses (Nadler *et al*, 1983; Otsuka *et al*, 2004). Thus, if our *in vitro* cytotoxicity data correctly predicts *in vivo* efficacy (which has yet to be determined), only a subpopulation of lymphoma patients would have tumours suitable for treatment with internalization-dependent anti-CD19 ADCs. This suggests that it may be worthwhile to examine biopsies from past and future trials for CD21 expression (as well as CD19 expression) to determine if CD21 levels do indeed predict patient response to internalization-dependent anti-CD19 ADC therapy.

In summary, we have shown that CD21 expression significantly retards the internalization of anti-CD19 antibodies and decreases the cytotoxicity of anti-CD19-MCC-DM1 conjugates. While CD21 expression may not be the only ‘resistance factor’ for anti-CD19-MCC-DM1 therapy, these data should aid the selection of suitable preclinical lymphoma xenograft models for testing this ADC, with CD21^−^ or CD21^lo^ models being expected to show greater efficacy. Our results should be generally applicable to other internalization-dependent anti-CD19 ADCs (Sapra & Allen, 2002). However, DM1 conjugation does not alter the internalization of anti-CD19 antibody (data not shown), whereas ricin conjugation to another anti-CD19 antibody increases its internalization, possibly via toxin dimerization and CD19 cross-linking (Goulet *et al*, 1997). Furthermore, since CD21 is only expressed in approximately two-third of B-cell lymphomas and only at low levels in half of those (Table I), our results may even be of clinical importance in selecting appropriate patients for anti-CD19 ADC therapy. Clearly, this is an avenue of future research worth investigating.

## Supplementary material

The following supplementary material is available for this article online:

Fig S1 The differential internalization of anti-CD19 between cell lines is not limited to the B496 antibody.

Fig S2 CD21 transfected into Ramos cells complexes with CD19.

Fig S3 Anti-CD19 is delivered to lysosomes in all internalizing cell lines.

Fig S4 Cytotoxicity experiment controls.

Fig S5 Dual IF of lymphomas reveals variability in CD21 expression.

Appendix SI Methods.

The material is available as part of the online article from: http://www.blackwell-synergy.com/doi/abs/10.1111/j.1365-2141.2007.06883.x.

(This link will take you to the article abstract.)

## References

[b1] American Cancer Society (2007). What is Non-Hodgkin’s Lymphoma?. Cancer Reference Information.

[b2] Austin CD, De Maziere AM, Pisacane PI, van Dijk SM, Eigenbrot C, Sliwkowski MX, Klumperman J, Scheller RH (2004). Endocytosis and sorting of ErbB2 and the site of action of cancer therapeutics trastuzumab and geldanamycin. Molecular Biology of the Cell.

[b3] Austin CD, Wen X, Gazzard L, Nelson C, Scheller RH, Scales SJ (2005). Oxidizing potential of endosomes and lysosomes limits intracellular cleavage of disulfide-based antibody-drug conjugates. Proceedings of the National Academy of Sciences of the United States of America.

[b4] Barrault DV, Knight AM (2004). Distinct sequences in the cytoplasmic domain of complement receptor 2 are involved in antigen internalization and presentation. Journal of Immunology.

[b5] Bonifacino JS, Traub LM (2003). Signals for sorting of transmembrane proteins to endosomes and lysosomes. Annual Review of Biochemistry.

[b6] Bradbury LE, Kansas GS, Levy S, Evans RL, Tedder TF (1992). The CD19/CD21 signal transducing complex of human B lymphocytes includes the target of antiproliferative antibody-1 and Leu-13 molecules. Journal of Immunology.

[b7] Chaouchi N, Vazquez A, Galanaud P, Leprince C (1995). B cell antigen receptor-mediated apoptosis. Importance of accessory molecules CD19 and CD22, and of surface IgM cross-linking. Journal of Immunology.

[b8] Cherukuri A, Cheng PC, Pierce SK (2001a). The role of the CD19/CD21 complex in B cell processing and presentation of complement-tagged antigens. Journal of Immunology.

[b9] Cherukuri A, Cheng PC, Sohn HW, Pierce SK (2001b). The CD19/CD21 complex functions to prolong B cell antigen receptor signaling from lipid rafts. Immunity.

[b10] Cohen JH, Fischer E, Kazatchkine MD, Lenoir GM, Lefevre-Delvincourt C, Revillard JP (1987). Expression of CR1 and CR2 complement receptors following Epstein-Barr virus infection of Burkitt’s lymphoma cell lines. Scandinavian Journal of Immunology.

[b11] Cvetkovic RS, Perry CM (2006). Spotlight on rituximab in non-Hodgkin lymphoma and chronic lymphocytic leukemia. BioDrugs.

[b12] Doronina SO, Toki BE, Torgov MY, Mendelsohn BA, Cerveny CG, Chace DF, DeBlanc RL, Gearing RP, Bovee TD, Siegall CB, Francisco JA, Wahl AF, Meyer DL, Senter PD (2003). Development of potent monoclonal antibody auristatin conjugates for cancer therapy. Nature Biotechnology.

[b13] Echeverri C, Fisher S, King D, Craig FE (2002). Immunophenotypic variability of B-cell non-Hodgkin lymphoma: a retrospective study of cases analyzed by flow cytometry. American Journal of Clinical Pathology.

[b14] Erickson HK, Park PU, Widdison WC, Kovtun YV, Garrett LM, Hoffman K, Lutz RJ, Goldmacher VS, Blattler WA (2006). Antibody-maytansinoid conjugates are activated in targeted cancer cells by lysosomal degradation and linker-dependent intracellular processing. Cancer Research.

[b15] Fearon DT, Carroll MC (2000). Regulation of B lymphocyte responses to foreign and self-antigens by the CD19/CD21 complex. Annual Review of Immunology.

[b16] Flavell DJ, Warnes SL, Bryson CJ, Field SA, Noss AL, Packham G, Flavell SU (2006). The anti-CD20 antibody rituximab augments the immunospecific therapeutic effectiveness of an anti-CD19 immunotoxin directed against human B-cell lymphoma. British Journal Haematology.

[b17] Fra AM, Williamson E, Simons K, Parton RG (1994). Detergent-insoluble glycolipid microdomains in lymphocytes in the absence of caveolae. Journal of Biological Chemistry.

[b18] Freeman CB, Magrath IT, Benjamin D, Makuch R, Douglass EC, Santaella ML (1982). Classification of cell lines derived from undifferentiated lymphomas according to their expression of complement and Epstein–Barr virus receptors: implications for the relationship between African and American Burkitt’s lymphoma. Clinical Immunology and Immunopathology.

[b19] Fremeaux-Bacchi V, Fischer E, Lecoanet-Henchoz S, Mani JC, Bonnefoy JY, Kazatchkine MD (1998). Soluble CD21 (sCD21) forms biologically active complexes with CD23: sCD21 is present in normal plasma as a complex with trimeric CD23 and inhibits soluble CD23-induced IgE synthesis by B cells. International Immunology.

[b20] Ghetie MA, Picker LJ, Richardson JA, Tucker K, Uhr JW, Vitetta ES (1994). Anti-CD19 inhibits the growth of human B-cell tumor lines *in vitro* and of Daudi cells in SCID mice by inducing cell cycle arrest. Blood.

[b21] Ghetie MA, Podar EM, Ilgen A, Gordon BE, Uhr JW, Vitetta ES (1997). Homodimerization of tumor-reactive monoclonal antibodies markedly increases their ability to induce growth arrest or apoptosis of tumor cells. Proceedings of the National Academy of Sciences of the United States of America.

[b22] Gloghini A, Carbone A (1993). The nonlymphoid microenvironment of reactive follicles and lymphomas of follicular origin as defined by immunohistology on paraffin-embedded tissues. Human Pathology.

[b23] Goulet AC, Goldmacher VS, Lambert JM, Baron C, Roy DC, Kouassi E (1997). Conjugation of blocked ricin to an anti-CD19 monoclonal antibody increases antibody-induced cell calcium mobilization and CD19 internalization. Blood.

[b24] Hamann PR, Hinman LM, Hollander I, Beyer CF, Lindh D, Holcomb R, Hallett W, Tsou HR, Upeslacis J, Shochat D, Mountain A, Flowers DA, Bernstein I (2002). Gemtuzumab ozogamicin, a potent and selective anti-CD33 antibody-calicheamicin conjugate for treatment of acute myeloid leukemia. Bioconjugate Chemistry.

[b25] James JS, Dubs G (1997). FDA approves new kind of lymphoma treatment. Food and Drug Administration. AIDS Treat News.

[b26] Khine AA, Firtel M, Lingwood CA (1998). CD77-dependent retrograde transport of CD19 to the nuclear membrane: functional relationship between CD77 and CD19 during germinal center B-cell apoptosis. Journal of Cellular Physiology.

[b27] Kovtun YV, Audette CA, Ye Y, Xie H, Ruberti MF, Phinney SJ, Leece BA, Chittenden T, Blattler WA, Goldmacher VS (2006). Antibody-drug conjugates designed to eradicate tumors with homogeneous and heterogeneous expression of the target antigen. Cancer Research.

[b28] Lopez-Matas M, Rodriguez-Justo M, Morilla R, Catovsky D, Matutes E (2000). Quantitative expression of CD23 and its ligand CD21 in chronic lymphocytic leukemia. Haematologica.

[b29] Macia E, Ehrlich M, Massol R, Boucrot E, Brunner C, Kirchhausen T (2006). Dynasore, a cell-permeable inhibitor of dynamin. Developmental Cell.

[b30] Nadler LM, Ritz J, Hardy R, Pesando JM, Schlossman SF, Stashenko P (1981). A unique cell surface antigen identifying lymphoid malignancies of B cell origin. Journal of Clinical Investigation.

[b31] Nadler LM, Anderson KC, Marti G, Bates M, Park E, Daley JF, Schlossman SF (1983). B4, a human B lymphocyte-associated antigen expressed on normal, mitogen-activated, and malignant B lymphocytes. Journal of Immunology.

[b32] van Oosterhout YV, van den Herik-Oudijk IE, Wessels HM, de Witte T, van de Winkel JG, Preijers FW (1994). Effect of isotype on internalization and cytotoxicity of CD19-ricin A immunotoxins. Cancer Research.

[b33] Otsuka M, Yakushijin Y, Hamada M, Hato T, Yasukawa M, Fujita S (2004). Role of CD21 antigen in diffuse large B-cell lymphoma and its clinical significance. British Journal Haematology.

[b34] Perrin-Cocon LA, Villiers CL, Salamero J, Gabert F, Marche PN (2004). B cell receptors and complement receptors target the antigen to distinct intracellular compartments. Journal of Immunology.

[b35] Petrie RJ, Schnetkamp PP, Patel KD, Awasthi-Kalia M, Deans JP (2000). Transient translocation of the B cell receptor and Src homology 2 domain-containing inositol phosphatase to lipid rafts: evidence toward a role in calcium regulation. Journal of Immunology.

[b36] Polakis P (2005). Arming antibodies for cancer therapy. Current Opinion in Pharmacology.

[b37] Polson AG, Yu SF, Elkins K, Zheng B, Clark S, Ingle GS, Slaga DS, Giere L, Du C, Tan C, Hongo JA, Gogineni A, Cole MJ, Vandlen R, Stephan JP, Young J, Chang W, Scales SJ, Ross S, Eaton D, Ebens A (2007). Antibody-drug conjugates targeted to CD79 for the treatment of non-Hodgkin’s lymphoma. Blood.

[b38] Press OW, Farr AG, Borroz KI, Anderson SK, Martin PJ (1989). Endocytosis and degradation of monoclonal antibodies targeting human B-cell malignancies. Cancer Research.

[b39] Press OW, Howell-Clark J, Anderson S, Bernstein I (1994). Retention of B-cell-specific monoclonal antibodies by human lymphoma cells. Blood.

[b40] Pulczynski S, Boesen AM, Jensen OM (1993). Antibody-induced modulation and intracellular transport of CD10 and CD19 antigens in human B-cell lines: an immunofluorescence and immunoelectron microscopy study. Blood.

[b41] Pulczynski S, Boesen AM, Jensen OM (1994). Modulation and intracellular transport of CD20 and CD21 antigens induced by B1 and B2 monoclonal antibodies in RAJI and JOK-1 cells – an immunofluorescence and immunoelectron microscopy study. Leukemia Research.

[b42] Sapra P, Allen TM (2002). Internalizing antibodies are necessary for improved therapeutic efficacy of antibody-targeted liposomal drugs. Cancer Research.

[b43] Schmid SL, McNiven MA, De Camilli P (1998). Dynamin and its partners: a progress report. Current Opinion in Cell Biology.

[b44] Scoazec JY, Berger F, Magaud JP, Brochier J, Coiffier B, Bryon PA (1989). The dendritic reticulum cell pattern in B cell lymphomas of the small cleaved, mixed, and large cell types: an immunohistochemical study of 48 cases. Human Pathology.

[b45] Sieber T, Schoeler D, Ringel F, Pascu M, Schriever F (2003). Selective internalization of monoclonal antibodies by B-cell chronic lymphocytic leukaemia cells. British Journal Haematology.

[b46] Smith MR (2003). Rituximab (monoclonal anti-CD20 antibody): mechanisms of action and resistance. Oncogene.

[b47] Tedder TF, Goldmacher VS, Lambert JM, Schlossman SF (1986). Epstein–Barr virus binding induces internalization of the C3d receptor: a novel immunotoxin delivery system. Journal of Immunology.

[b48] Tedder TF, Zhou LJ, Engel P (1994). The CD19/CD21 signal transduction complex of B lymphocytes. Immunology Today.

[b49] Tessier J, Cuvillier A, Glaudet F, Khamlichi AA (2006). Internalization and molecular interactions of human CD21 receptor. Molecular Immunology.

[b50] Uckun FM, Jaszcz W, Ambrus JL, Fauci AS, Gajl-Peczalska K, Song CW, Wick MR, Myers DE, Waddick K, Ledbetter JA (1988). Detailed studies on expression and function of CD19 surface determinant by using B43 monoclonal antibody and the clinical potential of anti-CD19 immunotoxins. Blood.

[b51] Vervoordeldonk SF, Balkenende AY, van den Berg H, von dem Borne AE, van der Schoot CE, Van Leeuwen EF, Slaper-Cortenbach IC (1996). Degradation of radioiodinated B cell monoclonal antibodies: inhibition via a FCgamma-receptor-II-mediated mechanism and by drugs. Cancer Immunology, Immunotherapy.

[b52] Vose JM (1999). Antibody-targeted therapy for low-grade lymphoma. Seminars in Hematology.

[b53] Watts C, Marsh M (1992). Endocytosis: what goes in and how?. Journal of Cell Science.

[b54] Wiels J, Fellous M, Tursz T (1981). Monoclonal antibody against a Burkitt lymphoma-associated antigen. Proceedings of the National Academy of Sciences of the United States of America.

[b55] Xie H, Audette C, Hoffee M, Lambert JM, Blattler WA (2004). Pharmacokinetics and biodistribution of the antitumor immunoconjugate, cantuzumab mertansine (huC242-DM1), and its two components in mice. Journal of Pharmacology and Experimental Therapeutics.

