# Supplementary material for: High CD21 expression inhibits internalization of anti-CD19 antibodies and cytotoxicity of an anti-CD19-drug conjugate
Source: Br J Haematol. 2007 Nov 7;140(1):46–58. doi: 10.1111/j.1365-2141.2007.06883.x (PMC2228374; doi:10.1111/j.1365-2141.2007.06883.x)

Ingle *et al*. Supplemental Figure 1: Three other anti-CD19 antibodies behave similarly to B496

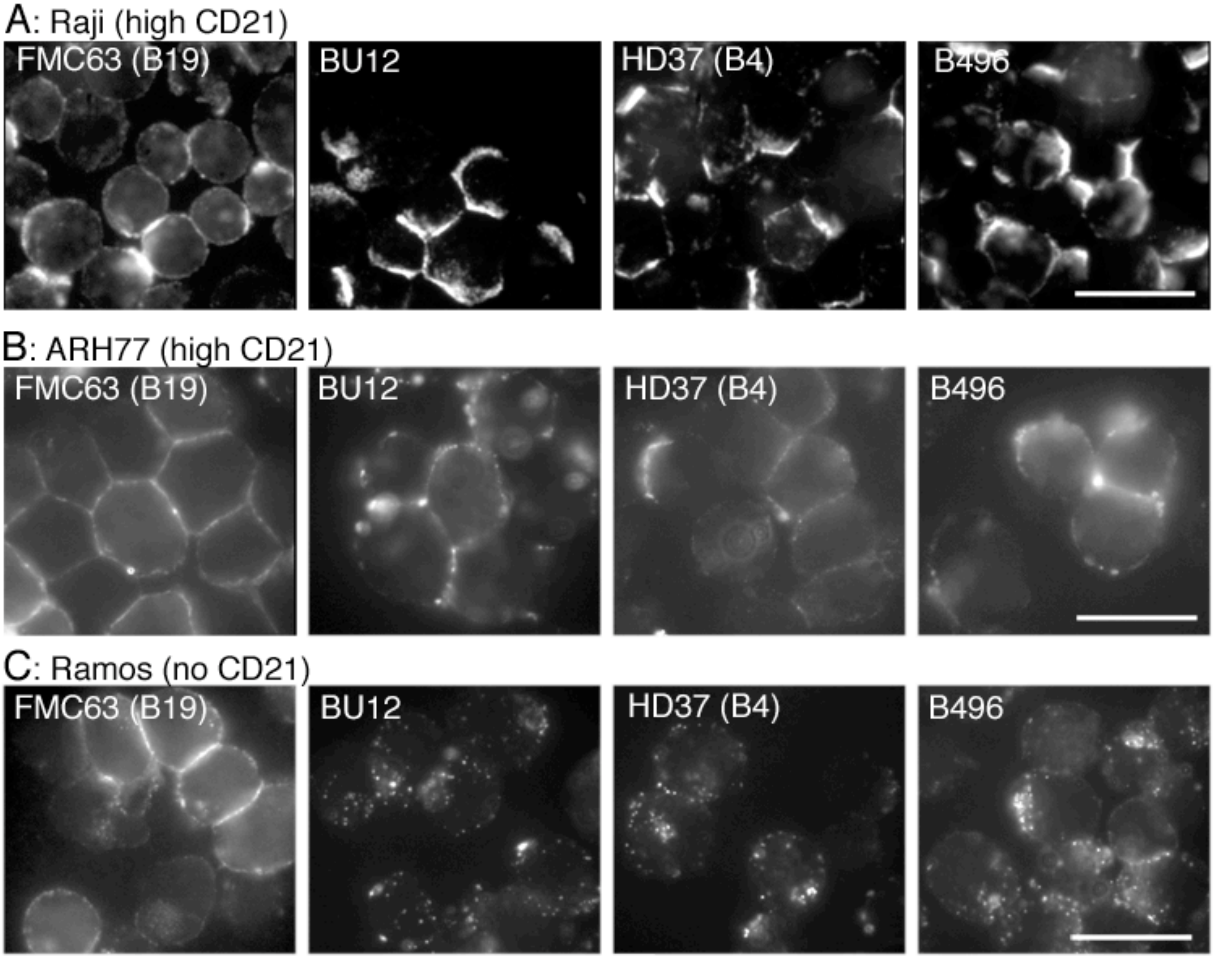

Ingle *et al*. Supplemental Figure 2:
CD21 complexes with CD19 in the Ramos-CD21 clone 1 stable cell line

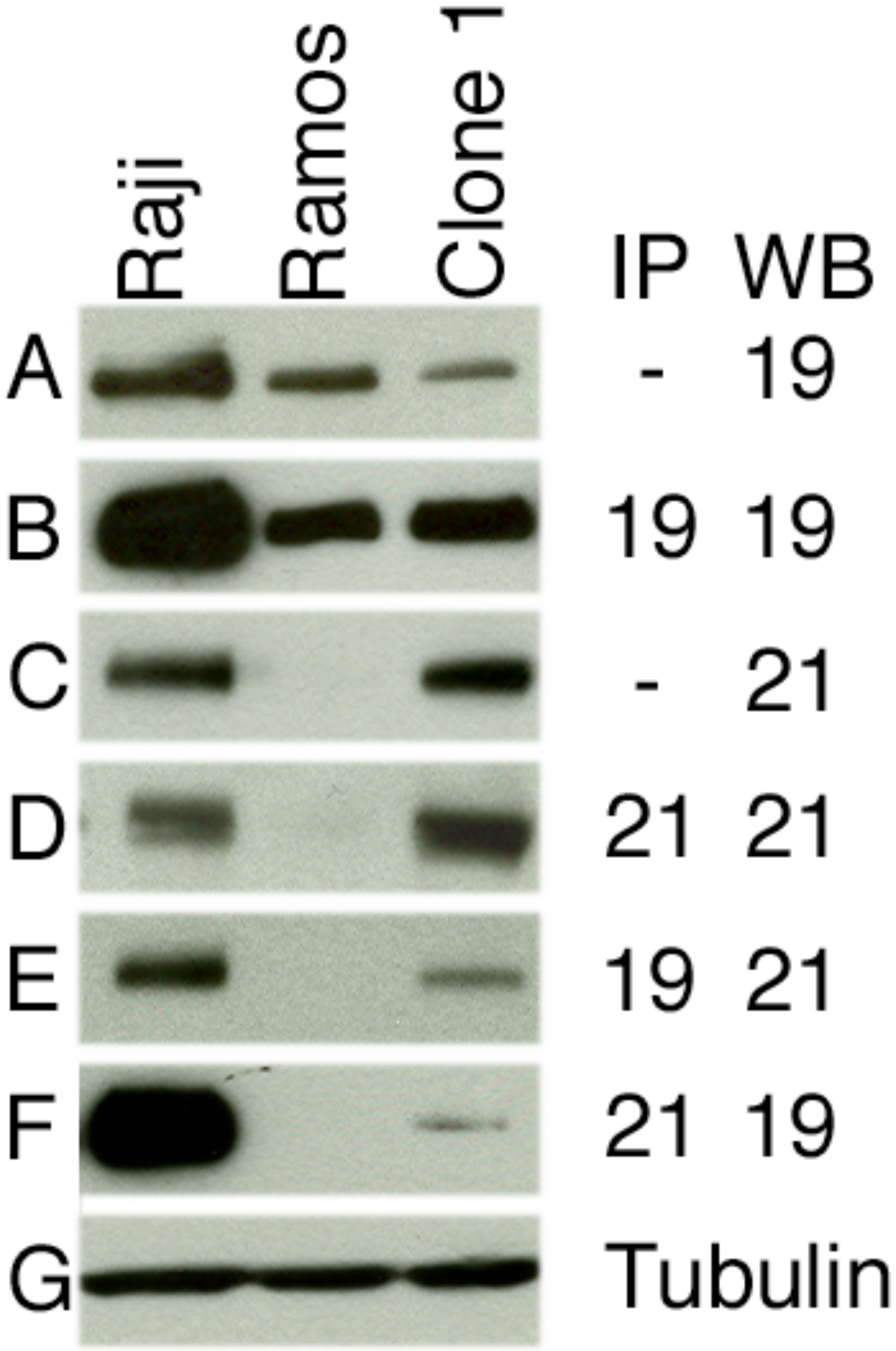

Ingle *et al*. Supplemental Figure 3: Anti-CD19 is delivered to lysosomes by 3 hours in all the internalizing cell lines

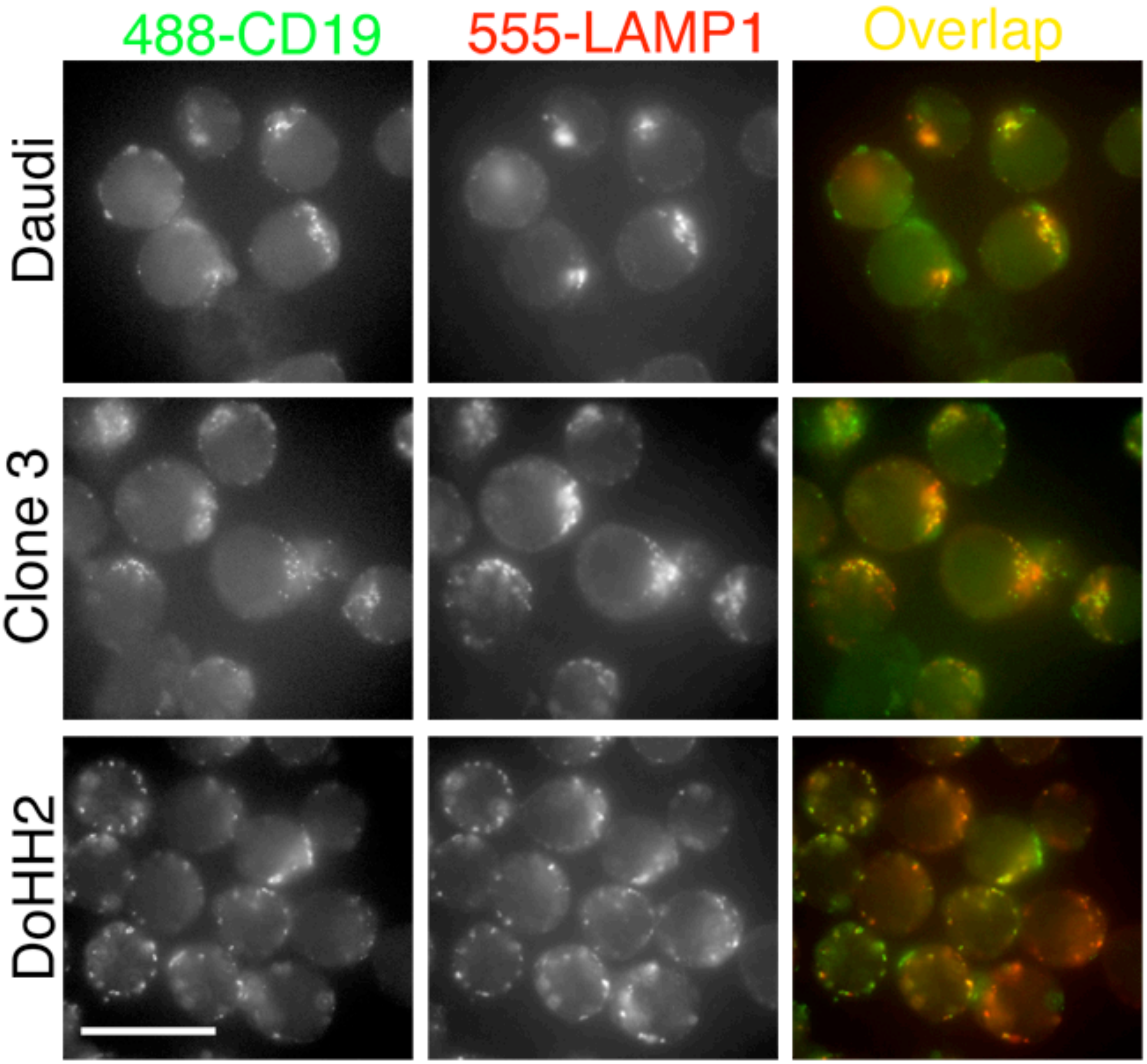

Ingle et al.
Supplemental Figure 4:
control cytotoxicity
assays

A) Viability assay of naked anti-CD19 antibodies in ARH77 cells

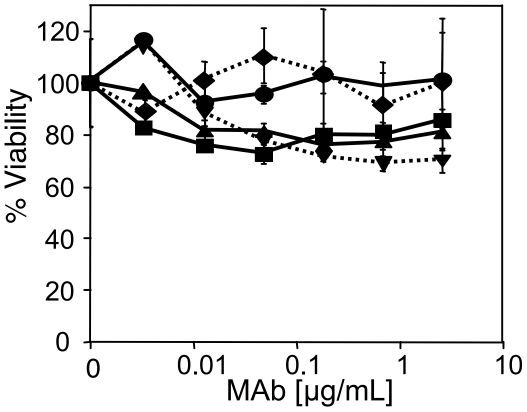

B) Apoptosis assay in ARH77 cells

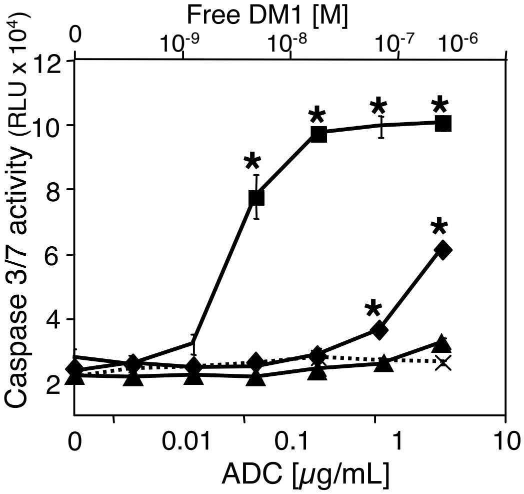

C) Anti-CD19-MCC-DM1 in Jurkat cells

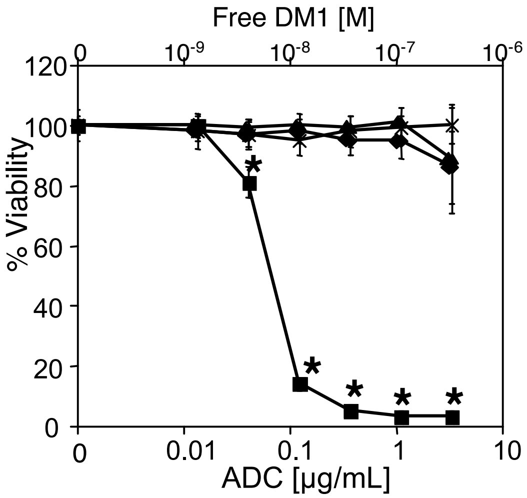

Ingle *et al*. Supplemental Figure 5:
Dual CD19,21 immunofluorescence of lymphomas

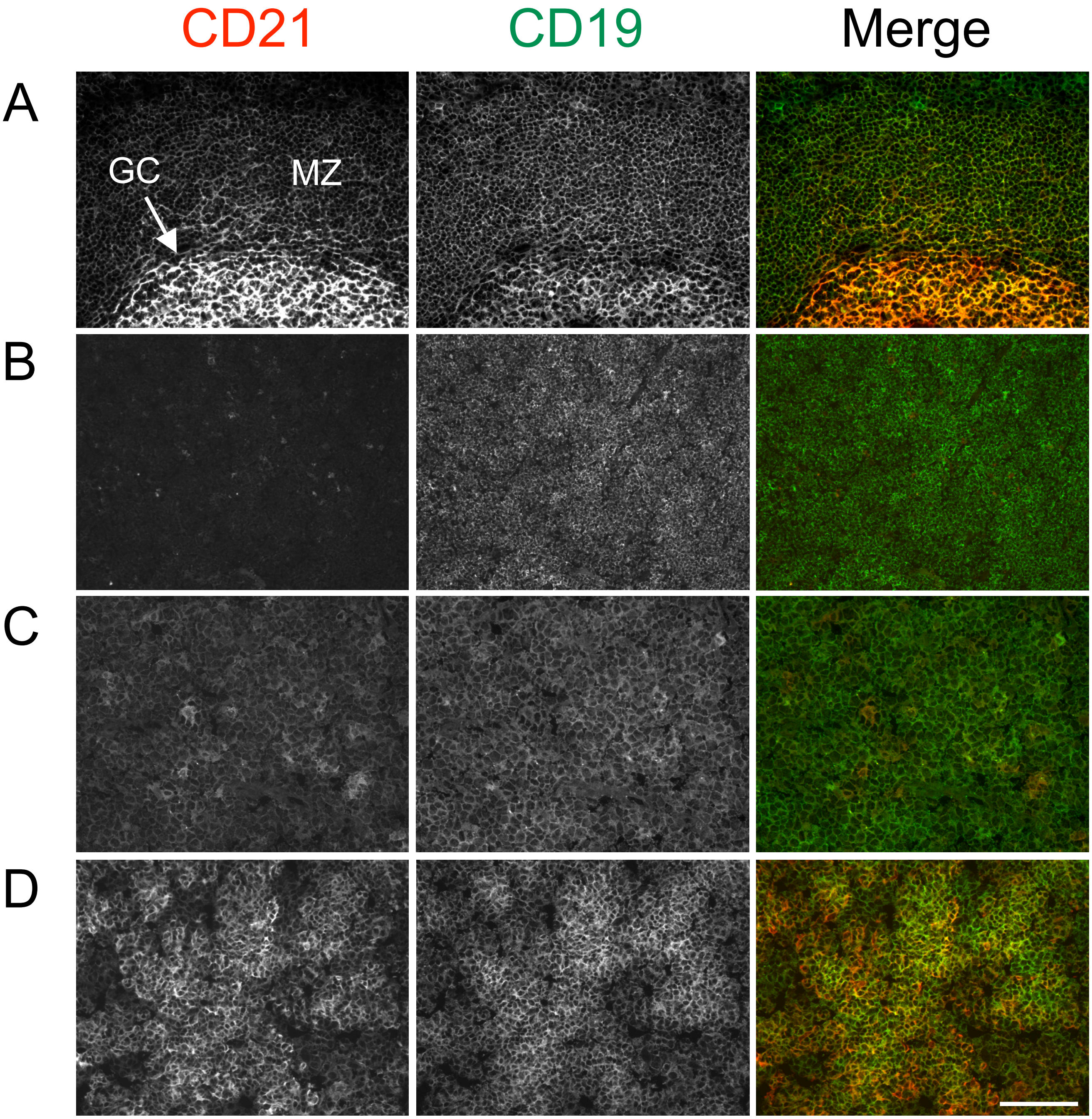

Supplement: Fig S1 — The differential internalization of anti-CD19 between cell lines is not limited to the B496 antibody. [file bjh0140-0046-SD1.pdf]
