# Supplementary material for: High CD21 expression inhibits internalization of anti-CD19 antibodies and cytotoxicity of an anti-CD19-drug conjugate
Source: Br J Haematol. 2007 Nov 7;140(1):46–58. doi: 10.1111/j.1365-2141.2007.06883.x (PMC2228374; doi:10.1111/j.1365-2141.2007.06883.x)
Supplement: Appendix SI — Methods. [file bjh0140-0046-SD6.doc]

**Supplemental information** for Ingle et al: High CD21 expression inhibits internalization of anti-CD19 antibodies and cytotoxicity of an anti-CD19-drug conjugate

**Supplemental Methods**

*CD19-CD21 co-immunoprecipitation*. 20 x 10^6^ B-cells were washed twice in PBS, resuspended in 1ml ice-cold digitonin buffer modified from Matsumoto *et al.* (Matsumo*to et a*l., 1991) (10 mM triethanolamine, 150 mM NaCl, 1 mM EDTA, 1 % digitonin, 1 mM PMSF, 1 µg/ml pepstatin A and 1x Complete Protease Inhibitors (Roche); sheared 12x with a 25G needle and rotated for 20 min at 4°C. After clearing the lysate by centrifugation and pre-adsorption on protein-G sepharose beads, 50 µl was added to sample buffer as the total lysate control. Equivalent protein concentrations (determined by the BCA kit (Pierce)) of the remainder were incubated with 10 µg anti-CD19 (HD37) or anti-CD21 (HB135) for 1 h, followed by 50 µl protein-G sepharose (50% (v/v) slurry in digitonin buffer) overnight at 4°C. Beads were washed 5x in digitonin buffer, 2x in 50 mM Tris, then boiled for 10 min in 50 µl 2x reducing sample buffer. 20 µl each sample was loaded on 4-20% Tris-Glycine SDS-PAGE gels and transferred to nitrocellulose. Blots were blocked in 5% (w/v) milk, 0.1% Tween-20 in PBS, cut into horizontal strips and probed with 1:1000 goat anti-CD21 (C-20, Santa Cruz Biotech), 1:1000 rabbit anti-CD19 **(**Cell Signaling Technology #3574) or 1: 5000 mouse anti-tubulin (clone 1A2, Sigma), followed by HRP-conjugated secondaries. Bands were captured on X-ray film following incubation with ChemiGlow enhanced chemiluminscence reagent (Alpha Innotech).

*Frozen lymphoma section immunofluorescence*. Frozen human lymphoma tissue samples from the Genentech Human Tissue Bank were sectioned at 5 µm onto microscope slides, air-dried and acetone-fixed for 5 min. After staining with hematoxylin and eosin to assess tissue quality and tumor grade (by pathologist H.K.), sections were incubated with 5 µg/ml both anti-CD19 (SJ25-C1, Southern Biotechnology, mouse IgG1) and anti-CD21 (HB135, mouse IgG2a), followed by 5 μg/ml both FITC-anti-mouse IgG1 and TRITC-anti-mouse IgG2a (Southern Biotechnology). Nuclei were counterstained with DAPI and sections coverslipped with ProLong Gold antifade (Molecular Probes). Slides were viewed with a 20x objective (or 10x in panel 5B) on an Olympus BX51 microscope and imaged with a Hamamatsu CCD camera using MetaMorph software (Molecular Devices). Figures were assembled using Adobe Photoshop CS.

Semi-quantitative assessments of CD19 and CD21 staining intensities in normal and lymphoma B-cells (excluding follicular dendritic cell staining) were made by a pathologist (H.K.), and expressed as negative, 1+ (weak), 2+ (moderate) and 3+ (strong).

**Supplemental reference**

Matsumoto, A.K., Kopicky-Burd, J., Carter, R.H., Tuveson, D.A., Tedder, T.F., and Fearon, D.T. (1991). Intersection of the complement and immune systems: a signal transduction complex of the B lymphocyte-containing complement receptor type 2 and CD19. J Exp Med *173*, 55-64.
